# Primary fibular grafting combined with double plating in distal femur fractures in elderly patients

**DOI:** 10.1007/s00264-022-05441-x

**Published:** 2022-05-17

**Authors:** Fady M. Ibrahim, Ahmed K. El Ghazawy, Mohammed A. Hussien

**Affiliations:** Department of Orthopedic Surgery, Faculty of Medicine, Ain Shams University, 38 Abbassia Square, Next to Al-Nour Mosque, Cairo, Egypt

**Keywords:** Distal femur fracture, Elderly, Double plating, Fibular graft

## Abstract

**Purpose:**

To report functional and radiological outcomes of using primary fibular graft together with double plating in distal femoral fractures in the elderly.

**Methods:**

A retrospective study on 30 elderly patients with comminuted distal femoral fractures managed by primary fibular grafting and double plating through an anterior midline approach has been conducted. Only isolated distal femoral fractures type 33-A3, 33-C2, and 33-C3 were included. The patient’s mean age was 75.3 years. Evaluation included operative time, blood loss, time to union, knee range of motion, Sanders scoring, and presence of complications.

**Results:**

The average follow-up period was 26.6 months. Mean intraoperative blood loss was 401 ml, and mean operative time was 216 min. All patients had a knee range of motion (90–120°) during follow-up. Time for union ranged from 16 to 23 weeks with a mean of 18.4 weeks, with no cases of non-union. A total of 22 patients (73.3%) showed excellent functional outcomes, and the remaining eight (26.7%) showed good functional outcomes according to the Sanders scoring system. Only two cases (6.6%) had superficial wound infections managed conservatively. No post-operative deformity, loss of reduction, or implant failure was observed until the end of follow-up period.

**Conclusion:**

Primary fibular grafting combined with double plating of comminuted distal femur fractures in patients above 70 years is an effective technique with higher rates of union and lower re-operation rates compared to other fixation modalities.

## Introduction

The incidence of elderly population sustaining distal femoral fractures had been increasing in the last two decades with a changing epidemiological pattern as regards female to male ratio (2:1) [1–4]. Distal femoral fractures compromise 3–6% of all femoral fractures [1, 5]. The most common mechanism of injury is low-energy trauma following a simple fall in this osteoporotic population [1, 6, 7]. Being comparable to proximal femoral fractures regarding high mortality and comorbidity [8], inadequate number of studies addressed the best fixation protocol, especially in this age group [9].

Surgical fixation yielded better results compared to conservative treatment [10]; nevertheless, many post-operative complications are encountered including prolonged hospital stay, DVT (6%), up to 8% early mortality rising to 25% late mortality rates within one year, and failure to return to the pre-fall level of activity especially in this age group to avoid being dependent [11–13].

Posing a major problem, nonunion after lateral plating alone was reported in up to 21% of the cases [14], many studies stated that metaphyseal comminution, poor bone quality, and inadequate fixation are the main causes for nonunion reflecting the necessity to deal with these problems [15, 16]. Tendency to fail in varus is another major problem, also in cases of comminution [15, 16]. Sanders et al. [17] emphasized that it is crucial to reestablish medial continuity to avoid collapse. Varus malunion leading to osteoarthritis reaches up to 50% at six year follow-up, causing high disability and may necessitate a total knee [18].

Using primary fibular grafting with stable rigid double plating fixation after anatomical reduction of the articular and metaphyseal fractures allowing an early range of motion and rehabilitation may decrease the reportedly high rates of nonunion, malunion, varus collapse, and the need for secondary surgery in this fragile population.

## Materials and methods

After Institutional Review Board approval, a retrospective study of elderly patients with distal femur fractures was conducted. We searched our medical records for elderly patients with distal femur fractures managed by double plating and primary fibular grafting technique.

Inclusion criteria included age over 70 years, displaced isolated distal femoral fracture with metaphyseal comminution (AO/OTA 33-A3, 33-C2, 33-C3) fractures.

Exclusion criteria were polytrauma patients, open fractures, pathological fractures, patients not fit for surgery, and non-ambulatory patients. Patients with less than two years of clinical and radiographic follow-up were also excluded from evaluation.

Patient charts and radiographs collected from the electronic medical record were reviewed for demographic data, AO/OTA classification, operative time, intraoperative blood loss, time to union, complications, reinterventions, and functional outcome scoring at the end of follow-up was evaluated according to the Sanders scoring system [17].

Thirty patients met the inclusion criteria between 2016 and 2019 and were enrolled in this study, 18 females and 12 males, with a mean age of 75.3 years. Informed written consent was taken regarding the study. Ten of the patients were classified as 33-A3, eight were 33-C3, and the majority were classified as 33-C2 (12 patients). The average time of surgery was 75 hours post-injury, and the main causes of delay were late presentation or adjustment of associated medical comorbidities. Demographic data of the studied patients are listed in Table 1.

**Table 1:** Age, sex, AO/OTA classification, and follow-up period

Patient	Age	Sex	AO/OTA classification	Follow-up period (month)
1	75	Female	33-C2	26
2	70	Female	33-A3	25
3	72	Male	33-A3	28
4	80	Female	33-C3	24
5	77	Male	33-C2	27
6	81	Male	33-C2	25
7	74	Female	33-A3	28
8	82	Male	33-C3	24
9	73	Female	33-A3	29
10	70	Female	33-C2	24
11	72	Male	33-C3	25
12	81	Female	33-C2	27
13	71	Male	33-A3	28
14	73	Female	33-C2	26
15	73	Female	33-A3	24
16	75	Male	33-C2	29
17	81	Female	33-C3	25
18	82	Male	33-C2	25
19	71	Female	33-C3	30
20	74	Female	33-A3	30
21	70	Male	33-C2	25
22	81	Male	33-C3	24
23	79	Male	33-C2	26
24	81	Female	33-A3	24
25	72	Female	33-C3	31
26	71	Female	33-C3	29
27	73	Male	33-A3	27
28	72	Female	33-C2	28
29	82	Female	33-C2	25
30	71	Female	33-A3	30

### Surgical technique

The procedure was done under combined spinal-epidural anaesthesia, except in five patients where general anesthesia was done due to failed spinal anaesthesia. The surgery was done on a translucent orthoapedic table in a supine position with a bolster underneath the knee. No tourniquet was used. One gram of third generation cephalosporins was given at induction and continued for two days post-operatively.

After standard prepping and draping of the whole limb, a midline anterior skin incision was done followed by either a lateral or a medial parapatellar approach according to the proximal extent of the lateral condyle fracture to facilitate lateral plate and screw positioning. First, anatomical reduction of the articular surface with preliminary wiring and interfragmentary screws is done, followed by reduction and fixation of medial cortex using L or T proximal tibial plate or distal femoral locked plate of the opposite side with two proximal and two distal screws. Then, direct anatomical reduction of the lateral condyle is done aiming for anatomical shaft/condyle relation regarding length, varus/valgus alignment aided by image intensifier. Completing the lateral fixation using long lateral distal femoral locked plate is done. Regarding the metaphyseal comminution gap created following anatomical reduction, harvested fibular graft together with preserved comminuted bony fragments is used to fill it.

Ipsilateral fibular graft harvest was done either using a standard protocol. A lateral incision over the fibula according to the desired length of the graft is made, keeping in mind to be 5 cm distal to the neck fibula proximally and 10 cm proximal to lateral malleolus distally to avoid complications. After fascial incision, retraction of peroneal muscles and incision of fibular periosteum is done. All muscles were subperiosteally dissected off using periosteum elevator, careful dissection is mandatory proximally to avoid damaging the peroneal nerve, then a bone saw is used to harvest the graft.

**Fig. 1:**
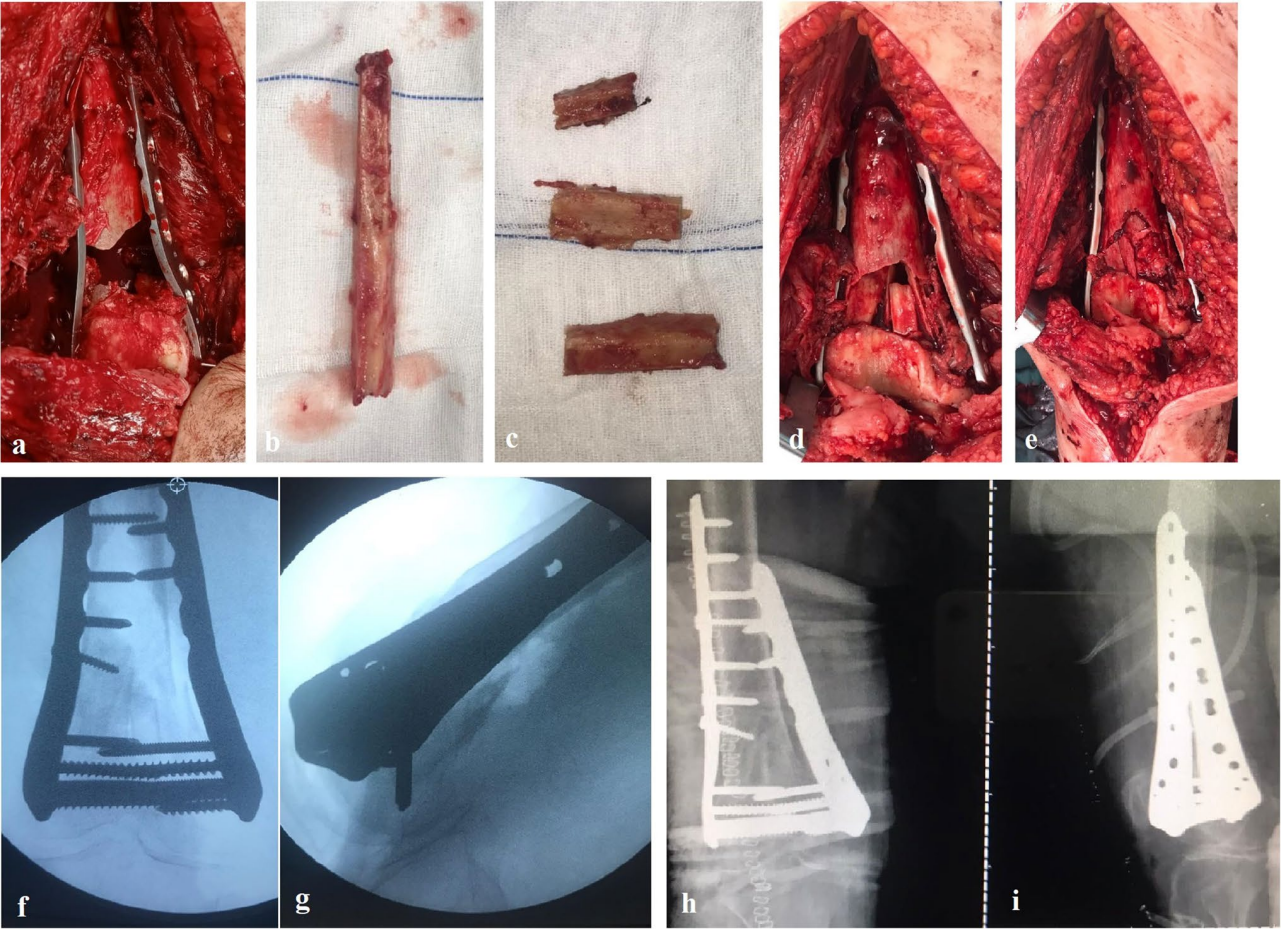
Female patient 70 years old, Sanders functional scoring 38 points with ROM 110°, no pain, and unrestricted walking at the end of the follow-up period (25 months). **a** Comminution gap following anatomical reduction. **b** Harvested fibular graft. **c** The fashioned graft according to gap size. **d** Lateral, medial, and posterior strut inserted. **e** Reconstruction of anterior cortex. **f** Intra-operative Ap image showing graft position, alignment. **g** Intra-operative lateral image showing alignment. **h** Post-operative Ap PXR showing anatomical reduction. **i** Post-operative Lateral PXR showing alignment

The harvested fibular graft is then fashioned to the desired number, size, and shape and inserted at the gap of metaphyseal comminution at three main sites, medial, lateral, and posterior reconstructing the three cortices (Fig. 1), while the anterior cortex is reconstructed using the comminuted fragments. Additional screws from both plates help in fibular positioning.

Closure of the extensor mechanism was done with knee flexed 90° in a watertight manner after drain insertion, followed by subcutaneous and skin closure in layers without tension.

Post-operatively, post-operative plain X-rays were done, and quadriceps strengthening exercises started immediately from the second post-operative day. An enhanced rehabilitation program with early range of motion using continuous passive motion (CPM) device gradually increased daily as tolerated reaching 90° at three weeks and progressing to the full range at six weeks compared to the other side. Assisted weight bearing on the sane limb was encouraged starting from the first week. Full weight-bearing was initiated at three months whether callus formation was evident or not. Hospital discharge protocol was based on achieving assisted weight-bearing and ROM.

#### Follow-up protocol

**Fig. 2:**
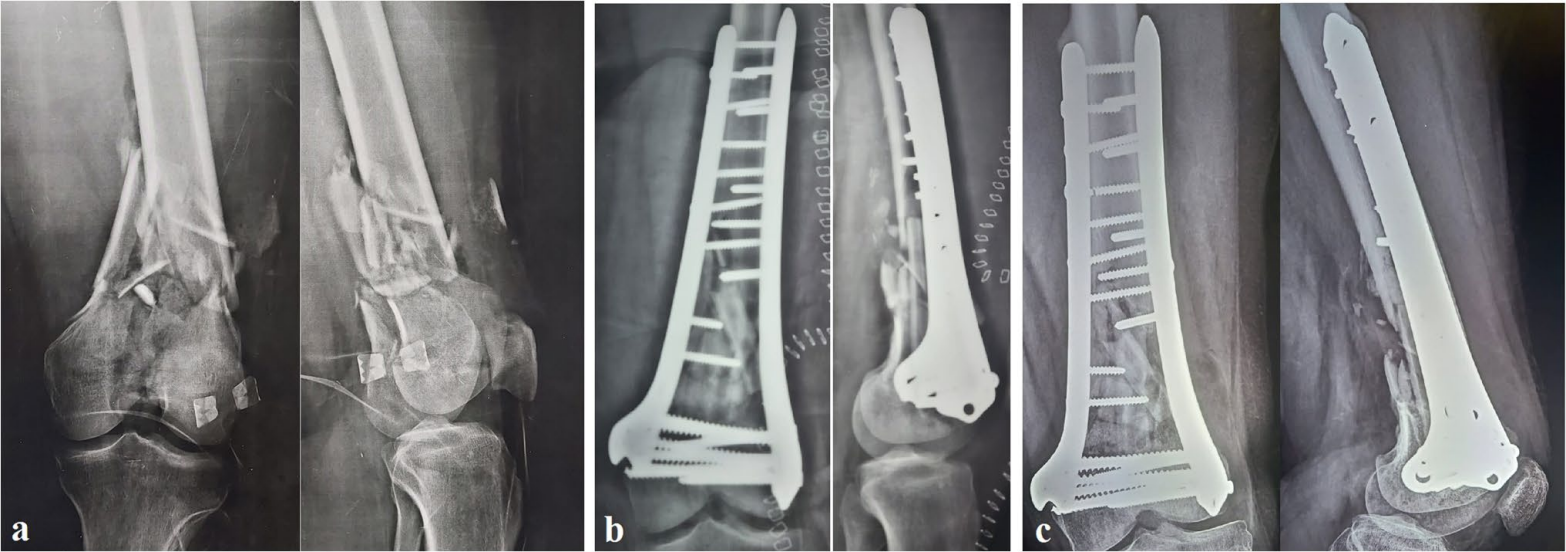
Male patient 82 years old, Sanders functional scoring 36 points with ROM 115°, no pain, and unrestricted walking at the end of the follow-up period (24 months). **a** Pre-operative AP/Lateral PXR showing a 33-C3 AO/OTA distal femur fracture with metaphyseal comminution. **b** Immediate post-operative PXRs showing anatomical reduction and fibular graft strutting 3 cortices. **c** Last follow-up PXRs show a fully united fracture with the incorporation of the fibular graft with no implant failure

**Fig. 3:**
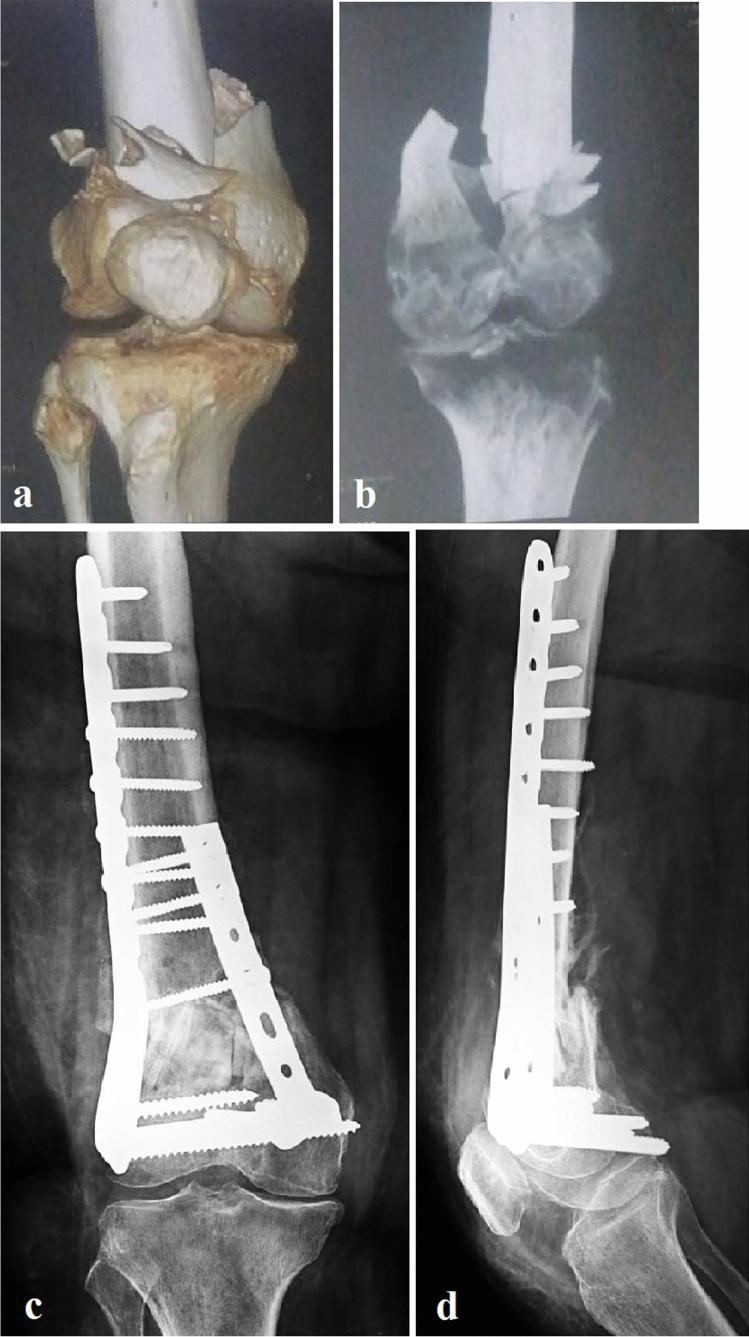
Male patient 75 years old, Sanders functional scoring 37 points with ROM 120°, no pain, and unrestricted walking at the end of the follow-up period (29 months). **a** Preoperative 3D CT scan showing a 33-C2 AO/OTA distal femur fracture. **b** Preoperative CT scan showing posterior comminution with intercondylar extension. **c** Last follow-up AP PXR showing anatomically reduced and fully united fracture. **d** Last follow-up Lateral PXR showing fully fused fibular graft

After their discharge, patients were followed up in the outpatient clinic at two weeks to ensure incision healing. Radiological follow-up in the first month was done every two weeks to assess any loss of reduction with early-assisted weight-bearing, followed by a plain X-ray each month for six months, and then every three months until the end of follow-up period (Fig. 2). Fracture union was defined by bridging callus at least in three out of four cortices and no pain clinically at fracture site (Fig. 3). In doubtful cases, a computed tomography scan was done. The mean follow-up period was 26.6 months.

## Results

The average intra-operative blood loss was 401 ml, and operative time ranged from 185 to 250 minutes (mean 216 min) **(**Table 2**)**. All patients had no postoperative knee stiffness with a range of motion (90–120°) during follow-up.

**Table 2:** Operative time, intra-operative blood loss, time to union (weeks), and Sanders scoring

Patient	Op. time (min)	Blood loss (ml)	Time to union (weeks)	Sanders scoring
1	200	320	16	38
2	190	490	18	38
3	210	320	22	36
4	240	550	23	36
5	220	300	16	30
6	190	380	17	32
7	210	400	16	36
8	230	340	20	36
9	235	510	18	38
10	245	270	22	37
11	250	360	19	35
12	240	300	18	36
13	190	430	16	34
14	200	490	17	38
15	195	420	21	38
16	205	410	19	37
17	250	570	16	38
18	185	350	20	36
19	235	370	18	40
20	200	450	16	40
21	200	550	17	34
22	245	340	19	34
23	205	300	16	34
24	210	330	21	36
25	235	490	20	38
26	225	460	18	32
27	215	300	22	38
28	225	520	18	40
29	210	400	17	38
30	190	310	16	40

The time for radiological union ranged from 16 to 23 weeks with a mean of 18.4 weeks, with no cases of non-union **(**Table 2). The majority (73.3%) of the enlisted patients showed excellent functional outcomes (36–40 points), and the remaining (26.7%) showed good functional outcomes (26–35 points) according to Sanders scoring system.

As for post-operative complications, only two cases (6.6%) were treated for superficial infection by antibiotics and daily dressing and did not require any further management. No post-operative deformity, loss of reduction, or implant failure was observed in our study population until the end of follow-up period.

## Discussion

Ongoing studies are being done on optimal management of distal femoral osteoporotic fractures in elderly, as fractures ultimately lead to exacerbation of underlying comorbidities, resulting in increased mortality. Faced by reduced healing and remodeling ability, challenges of fixation and attaining union in this population have been reported by several studies [15, 19], with nonunion rates up to 25% and implant failure rates up to 16% in some recent reports of locked lateral plating alone [15, 19, 20]. Peschiera et al. [20] in their study to detect predictive factors for fixation failure in distal femoral fractures found that lack of bone support with metaphyseal comminution, malalignment, and inadequate fixation were the main causes.

Dual plating was proven by several investigators to provide superior stability by decreasing the lever arm acting on femoral axis thus lowering applied load on the fracture [21–23]. Also, stabilizing both columns and providing a stronger fixation in osteoporotic comminuted distal femur fractures [24]. In a recent biomechanical study, artificial femora simulating osteoporotic bone with fixed distal femoral fractures were subjected to axial, torsional, and quasi-static loading, and dual plating showed significantly lesser longitudinal and shear displacement than single plating [25]. Another earlier biomechanical study by Prayson et al. [26] on synthetic bone stated that in highly comminuted metaphyseal fractures, supplementing the medial column by medial plating is highly recommended to prevent varus collapse.

Using Hak et al. [27] and Lujan et al. [28] recommendations regarding the effect of a large comminution gap in decreasing callus formation, primary grafting can abolish that effect and promote healing, thus reduces incidence of implant failure and the need for secondary intervention. Zlowodzki et al. [29] also documented that performing primary grafting is better in cases with severe comminution and stated that delaying secondary bone grafting was the main cause for fixation failure.

To the best of the author’s knowledge, no previous studies discussed using primary fibular grafting combined with double plating technique for distal femoral fractures in this age group, added to that the paucity of available literature discussing double plating in distal femur fractures.

In the current study, anterior midline approach was used for fracture reduction and fixation, and it allowed better visualization, especially the intraarticular extension, avoiding malalignment, and better assessment of comminution gap to determine size of needed fibular graft. Also, if total knee replacement is needed, it can be done through the same approach.

The autogenous fibular graft served several purposes during our surgical technique. Once in situ, the fibula acted as a bone substitute to fill the metaphyseal void, bony strut supporting medial and lateral cortices, and additional cortices enhancing screw fixation. Full weight-bearing was allowed after nearly 3 months not restricted to bony union as being a cortical strut; the fibula compared to iliac graft used in previous studies had no potential for resorption. Afterward, the fibula was used to assess progression of union and alignment. Adding primary bone graft at the metaphyseal comminution gap was a cornerstone for attaining an earlier and secure union abolishing the need for a second intervention as delayed grafting in this fragile population.

Our results are highly comparable to previous studies in operative time, blood loss, and postoperative range of motion, but superior in an earlier full weight-bearing at three months and union period around 18 weeks with better functional outcome scoring, with no cases of delayed or nonunion. Also dealing with only elderly patients, the results are highly promising for primary using fibular graft in these fractures.

Eight studies used primary bone grafting harvested from iliac crest or bone graft substitutes along with dual plating non used primarily fibular graft [17, 21–23, 30–33]; the patients enlisted in these studies were mostly young or middle-aged, except for Metwaly et al. [30] who recruited only the geriatric distal femur fracture with age > 60 years, and in Steinberg et al. [23] study, where the mean age was 76 years indicating the predominance of elderly population. On the contrary, this study enrolled only elderly patients above 70 years. Of those 8 studies, the first to report medial plating with an acceptable union rate was Sander’s et al. [17] in 1991, but only 9 patients were enrolled. Chapman et al. [31] in 1999 treated femoral non-union by double plating and grafting. Ziran et al. [21] in 2002 reported lateral and anterior plating at right angles to each other using anterior approach, and only 24 out of 36 patients united within 4 months. Holzman et al. [32] in 2016 reported adding iliac graft and medial plate in 23 non-united cases fixed with lateral plating alone, with only 21 cases reaching bony union at the end of the study. Steinberg et al. [23] published double plating technique using double approach for 32 patients including acute fractures, non-unions, and periprosthetic fractures, only 30 cases united within 12 months. Metwaly et al. [30] in 2018 reported a larger cohort of 23 elderly cases via a single incision double plating achieving 82.6% union rate within 9 months which is nearly doubled duration in comparison to our study, also 4 patients (17.4%) needed a secondary grafting operation for delayed union. Two studies enrolled only type C3 distal femur fractures fixed using double plates, Khalil et al. [22] in 2012 used a modified Olerud extensile approach to treat 25 patients, reaching bony union at around 18.3 weeks and Imam et al. [33] in 2018 treated 16 cases and reported achieving primary bone union at around 6 months in all cases except 1 which needed re-grating, with good functional outcome in only 68% of cases.

To sum up, our findings support that primary fibular grafting combined with dual plating of comminuted distal femur fractures in patients above 70 years is an effective technique with higher rates of union and lower reoperation rates compared to other fixation modalities. Additional biomechanical studies and randomized controlled trials are needed to further investigate the significance of this method.

## Conclusion

Primary fibular grafting combined with dual plate fixation of distal femur fractures in elderly patients does appear to be a safe, easy, and effective technique, and probably provides an improved healing environment for these fractures.

## Data Availability

The datasets generated during and/or analyzed during the current study are available from the corresponding author on reasonable request.
